# Disseminated *Mycobacterium abscessus* infection as a cause of autosensitization

**DOI:** 10.1016/j.jdcr.2021.08.002

**Published:** 2021-08-18

**Authors:** Margit Lai Wun Juhasz, Nathan W. Rojek, Catherine Diamond, Dan Mandel, Janellen Smith

**Affiliations:** a Department of Dermatology, University of California, Irvine, California; b Division of Infectious Diseases, Department of Medicine, University of California, Irvine, California; c Division of Rheumatology, Department of Medicine, University of California, Irvine, California

**Keywords:** autosensitization, hypersensitivity reaction, id reaction, mycobacterium, MAI, *Mycobacterium avium-intracellulare*

## Introduction

Id reactions (also known as autosensitization or autoeczematization reactions, or disseminated secondary eczema) are defined as skin lesions, often eczematous in appearance, in areas distant from the primary site of involvement, which can be due to other causes of skin inflammation, such as stasis dermatitis or allergic contact dermatitis, as well as infection.^1^ Few cases of autosensitization have been reported secondary to underlying *Mycobacterium* infection. In this report, we discuss a case of autosensitization secondary to disseminated *Mycobacterium abscessus* in a patient with underlying immunodeficiency.

## Case report

**Fig 1:**
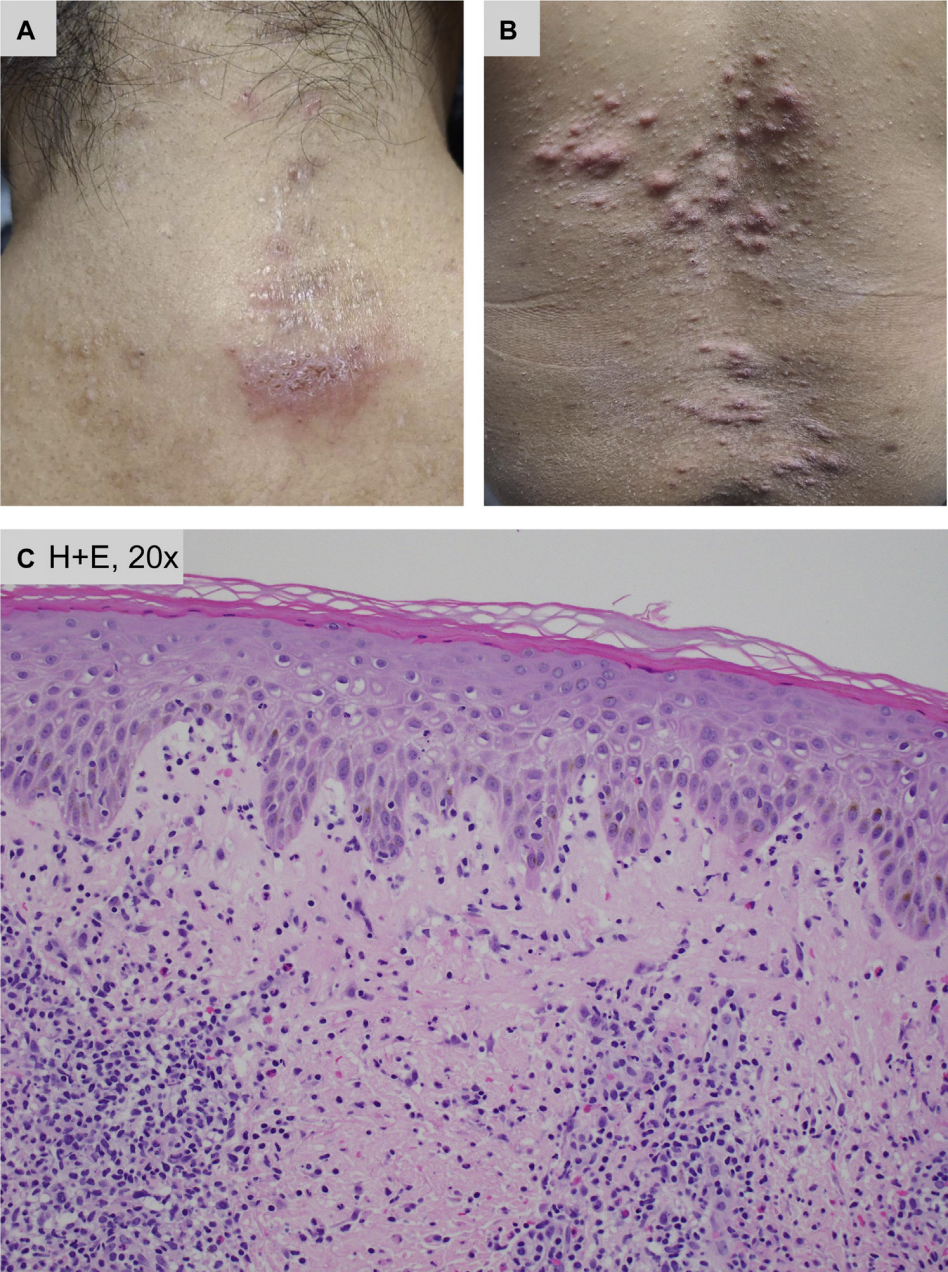
**A**, Our patient presented with ill-defined, finely scaly, thin plaques on the back and left lower extremity, which then spread over the entire body. **B**, Over time, the lesions became more papular in nature, but never became necrotic. All lesions were associated with intense pruritus. **C**, Hematoxylin-eosin histology from the second skin biopsy demonstrated spongiotic dermatitis with numerous eosinophils, which could be consistent with an autosensitization reaction. (**C**, Hematoxylin-eosin stain; original magnification: **C**, ×20.)

A 50-year-old Filipino woman with a past medical history of disseminated *Mycobacterium avium-intracellulare* (MAI) infection with associated erythema nodosum treated with 2 courses of triple antibiotic therapy 4 years ago, IgG lambda monoclonal gammopathy, mixed connective tissue disease, synovitis, acne, pustulosis, hyperostosis, osteitis syndrome with associated leukocytoclastic vasculitis and Ig A-related nephropathy, and breast cancer status post lumpectomy and radiation presented to the dermatology clinic with pink-to-brown, edematous and scaly, coin-shaped thin plaques associated with intense pruritus on the left lower leg and back, with subsequent spread over the body (Fig 1, *A* and *B*). HIV and syphilis serologies were negative. The patient was prescribed triamcinolone 0.1% ointment twice daily, instructions for a sensitive skin care regimen (including stopping all current over-the-counter topicals), and discontinuation of unnecessary oral medications and supplements for presumed eczematous dermatitis. Given no improvement after 1 month, a skin biopsy was performed and revealed spongiotic vesicular dermatitis with eosinophils. The patient was diagnosed with suspected eosinophilic dermatosis of hematologic malignancy given her history of monoclonal gammopathy and started on narrow-band ultraviolet B phototherapy twice weekly, cetirizine 10 mg oral twice daily, hydroxyzine 25 mg oral at night as needed for itch, as well as pramoxine and camphor/menthol lotion as needed for itch; in addition, topical steroid strength was increased to clobetasol 0.05% ointment twice daily. Despite extensive therapy for 7 months, the patient did not improve. She was started on oral methotrexate 15 mg weekly with folate supplementation by rheumatology and hematology/oncology, and long-term topical steroids were discontinued in favor of tacrolimus 0.1% ointment.

After 6 months of systemic immunosuppression, the patient's skin lesions worsened, making a continued eosinophilic dermatosis of hematologic malignancy unlikely. Due to an elevated absolute eosinophil count of 1.6 × 10^3^/μL, she was empirically treated with 1 dose of oral ivermectin 0.2 mg/kg for possible scabies infestation with no clinical improvement. Stool ova and parasite tests were negative. A second skin biopsy demonstrated superficial and deep perivascular infiltrate with numerous eosinophils; the differential diagnosis of such histopathology is broad and can include a primary spongiotic process, contact dermatitis, and dermal hypersensitivity (including autosensitization, drug, or arthropod assault) (Fig 1, *C*).

During a routine positron emission tomography scan to monitor her monoclonal gammopathy, the patient was noted to have widespread new lymphadenopathy. Biopsy of the right cervical lymph node was negative for malignancy, but tissue culture revealed *M* *abscessus subspecies abscessus*. Fite staining of her skin biopsy was negative for organisms, suggesting her skin lesions were not directly a result of *M* *abscessus* infiltration. The patient was referred to infectious disease where she was treated with 3 months of intravenous cefoxitin and amikacin. She took oral azithromycin for the first month of treatment but later switched oral omadacycline for months 2 and 3 due to macrolide resistance. Her dermatitis clinically improved subsequently, suggesting an autosensitization reaction to her underlying *M* *abscessus* infection. She is currently completing a 9- to 12-month course of oral clofazimine and omadacycline per infectious disease.

The patient has also now been diagnosed with interferon gamma antibody–related immunodeficiency which may have predisposed her to multiple disseminated mycobacterial infections. In addition, disseminated mycobacterial infections have been associated with synovitis, acne, pustulosis, hyperostosis, osteitis syndrome–like presentation,^1^ which may be a confounding factor in the patient's initial rheumatologic diagnosis.

## Discussion

Although the exact pathogenesis of autosensitization reactions is unknown, they are often associated with underlying stasis dermatitis and allergic contact dermatitis, with more than 60% of patients developing autosensitization. Regarding infectious causes, the most common is tinea pedis. Hypotheses regarding autosensitization include hematogenous dissemination of allergenic or microbial products, as well as activation of circulating memory T cells. Autosensitization reactions are treated similarly to other eczematous processes, including the use of topical corticosteroids, topical calcineurin inhibitors, phototherapy, immunosuppressive therapies, such as systemic corticosteroids, and symptomatic management of pruritus with oral antihistamines. For complete resolution, the underlying cause of autosensitization should be addressed.^2^

After a thorough literature search, only 2 cases reported autosensitization reactions to *Mycobacterium*—1 patient developed a generalized sarcoidal dermatitis to *Mycobacterium leprae* and another after Bacillus Calmette-Guerin immunotherapy for bladder cancer.^3^^,^^4^ More commonly reported hypersensitivity reactions to *Mycobacterium* include papulonecrotic or nodular tuberculid associated with *Mycobacterium tuberculosis*, MAI, and Bacillus Calmette-Guerin vaccine, as well as erythema induratum related to *M* *tuberculosis*.

Given our patient's biopsy-proven *M* *abscessus* infection affecting multiple lymph node sites and eczematous-appearing dermatitis, it was determined that her clinical presentation was most consistent with an autosensitization reaction. Improvement with antimycobacterial therapy further supports the hypothesis that her skin lesions were indeed due to the underlying infection. Interestingly, given our patient's history of disseminated MAI and *M* *abscessus* infection, it is possible that her prior leukocytoclastic vasculitis was a manifestation of an underlying *Mycobacterium* infection rather than her connective tissue disease, as previously thought.^5^

## Conflicts of interest

None disclosed.
